# Comparison of markerless and marker-based motion capture of gait kinematics in individuals with cerebral palsy and chronic stroke: A case study series

**DOI:** 10.21203/rs.3.rs-2557403/v1

**Published:** 2023-02-08

**Authors:** Emily A. Steffensen, Fabrício Magalhães, Brian A. Knarr, David C. Kingston

**Affiliations:** University of Nebraska at Omaha; University of Nebraska at Omaha; University of Nebraska at Omaha; University of Nebraska at Omaha

**Keywords:** Clinical gait, Joint Angle, Markerless, Walking, Overground

## Abstract

**Background:**

Three-dimensional (3D) motion analysis is an advanced tool used to quantify movement patterns in adults with chronic stroke and children with cerebral palsy. However, gold-standard marker-based systems have limitations for implementation in clinical settings. Markerless motion capture using *Theia3D* may provide a more accessible and clinically feasible alternative, but its accuracy is unknown in clinical populations. The purpose of this study was to quantify kinematic differences between marker-based and markerless motion capture systems in individuals with gait impairments.

**Methods:**

Three adults with chronic stroke and three children with cerebral palsy completed overground walking trials while marker-based and markerless motion capture data were synchronously recorded. Time-series waveforms of 3D ankle, knee, hip, and trunk angles were stride normalized and compared. Root mean squared error, maximum peak, minimum peak, and range of motion were used to assess discrete point differences. Pearson’s correlation and coefficient of multiple correlation were computed to assess similarity between the time series joint angle waveforms from both systems.

**Results:**

This study demonstrates that markerless motion capture using *Theia3D* produces good agreement with marker-based in the measurement of gait kinematics at most joints and anatomical planes in individuals with chronic stroke and cerebral palsy.

**Conclusions:**

This is the first investigation to study the feasibility of *Theia3D* markerless motion capture for use in chronic stroke and cerebral palsy gait analysis. Our results indicate that markerless motion capture may be an acceptable tool to measure gait kinematics in clinical populations to provide clinicians with objective movement assessment data.

## Background

Three-dimensional (3D) motion capture of human movement is a commonly used tool by biomechanists and clinicians to evaluate gait and mobility disorders.(1) Motion capture can objectively quantify segmental movement, joint range of motion, and spatiotemporal parameters.(2) Research in individuals post-stroke has extensively used 3D marker-based systems for measuring gait kinematics to better understand pathological movement patterns and inform rehabilitation strategies.(3–6) This is because stroke is a leading cause of long-term disability, is widely heterogeneous, and survivors often have significant mobility impairment. (7),(8) Similarly, clinical gait analyses use 3D kinematic outcomes as a key evaluation tool to guide surgical treatment and rehabilitation in children with cerebral palsy (CP).(9) Because of the unique presentations between heterogeneous patients, quantitative evaluation of movement patterns using 3D kinematics is critical for developing therapeutic interventions and evaluating function following corrective surgery.(9)

Marker-based motion capture poses several practical limitations that hinder the adoption of such techniques in clinical settings. As a result, there is limited accessibility to clinicians treating individuals post-stroke or with CP.(1, 9) Access to laboratories, financial cost and space requirements for equipment, and the need for highly trained personnel to physically palpate anatomical landmarks and operate equipment are noted issues.(1) When using marker-based motion capture systems, reflective markers are placed at anatomical landmarks, which requires minimal or tightly fitting attire and significant time of the participant for preparation and system calibration. Marker-based preparations can be challenging for clinical populations with limited endurance and sensory sensitivities common in those with stroke or CP.(10–12) Precise palpation of anatomical landmarks for marker placement is critical for accurate kinematic analysis; however, children with CP can present with cognitive disorders that make physical palpation and following instructions difficult for marker-based motion capture.(11–13) Due to these limitations, clinicians often rely on observational gait analysis scales and physical examination, which are more subjective.(1)

Markerless motion capture techniques have emerged as an alternative method for quantifying movement patterns that overcome many constraints of traditional marker-based systems. *Theia3D* (Theia Markerless, Inc, Kingston, ON), a commercially available and sophisticated markerless motion capture technology, uses deep learning algorithms to identify human anatomical features and estimate 3D position.(14) Because markerless systems use multiple 2D video cameras to track human movement patterns, they do not need reflective markers placed on the skin. This substantially reduces the burden of marker placement and clothing restrictions on participants, removing practical barriers to kinematic analysis for clinicians. This technology can be used with relatively low-cost 2D video cameras compared to infrared cameras needed for marker-based systems and does not require computer programming knowledge, or data processing time with some manufactures, by the clinical team. Therefore, markerless technology could increase the accessibility of quantitative gait analysis to clinical populations by removing several practical barriers of marker-based systems.(15, 16)

Previous work has highlighted the accuracy and reliability of *Theia3D*’s markerless technology in healthy populations, but verification has not yet been performed in clinical populations that present with gait and anatomical abnormalities. Investigations comparing an earlier version of this markerless software and marker-based motion capture from 30 healthy adults reported joint center estimates below 2.5 cm error for all major body joints (hip 3.6 cm).(16) In addition, lower limb segment angle errors were below 5.5° for all limbs (except transverse plane) with sagittal plane knee angles at 3.2° error in healthy adult gait.(15–17) *Theia3D*, has also proven reliable for measuring gait kinematics and spatiotemporal parameters from 55 healthy adults.(15–17) However, the accuracy of these systems in clinical populations, such as individuals post-stroke and children with CP, must be established for this technology to be translated to clinical settings and used to guide clinical treatment and rehabilitation.

The purpose of this study was to assess the accuracy of *Theia3D* markerless motion capture compared to a marker-based approach in the gait of those with CP and chronic stroke. We hypothesized that the kinematic analysis using *Theia3D* markerless motion capture would produce clinically acceptable results compared to gold standard marker-based motion capture. Specifically, we examined joint angles of the ankle, knee, hip, and trunk-pelvis angles in the sagittal, frontal, and transverse planes during overground walking at self-selected walking speed.

## Methods

### Study design

The aim of this study was to assess the accuracy of *Theia3D* markerless motion capture compared to a marker-based approach in CP and chronic stroke gait. This study was a case series; therefore, convenience sampling was used. A case series approach was used to demonstrate the ability of *Theia3D* to appropriately capture kinematics among very different clinical presentations of those with CP and chronic stroke. See Table 1 for clinical presentation and demographics of the participants.

### Participants

Ambulatory individuals between the age of 6–68 years old with neurological impairments were included. Three participants with CP (2M:1F; age 9.67 (4.73) years; height 1.42 (0.24) m; mass 39.13 (8.22) kg; Gross Motor Function Classification System (GMFCS) 1, 2, and 3) and three participants with chronic stroke (1M:2F; 7.02 (6.85) years since stroke; age 60.67 (11.02) years; height 1.63 (0.10) m; mass 82.83 (14.96) kg) were included. Exclusion criteria were reporting pain or any other disabling condition limiting task performance. This study was approved by the University of Nebraska Medical Center’s Institutional Review Board, and participants (or legal guardians) gave informed written consent prior to participation.

### Data Collection Procedure

Following informed consent, fifty-four reflective markers were affixed to the participants’ trunk, pelvis, and both thighs, shanks, and feet, as described in Appendix A. First, a 5-second static trial was collected. Participants were then asked to ambulate throughout the capture volume (length: 5m, width: 2m, high: 2m) at their self-selected pace. A 20-camera system was used to simultaneously capture both marker (10 Oqus 5 + cameras, 100 Hz, Qualisys AB, Gothenburg, Sweden) and video (2 Miqus and 8 Miqus Hybrid cameras, 80 Hz, Qualisys AB, Gothenburg, Sweden) data using the Gait Project Automation Framework (PAF) analysis module in Qualisys Tracking Manager (QTM, version 2021.2, Qualisys AB, Gothenburg, Sweden). Three walking trials were performed where two full strides (four steps) were collected for each limb per trial.

### Data Analysis

After recording static and walking trials, the Qualisys Gait PAF was used to export video data to Theia 3D (2022.1.0.2309 [patch: 7], Theia Markerless, Inc.). The configuration settings used in Theia 3D for model creation included: Render Smooth IK, Enable 3 DOF Knee, and GCVSL Filter Cutoff Freq: 5 Hz. The trajectories from reflective markers were tracked and labeled; then, marker-based and markerless approaches exported separate .c3d files used for kinematic analysis.

Marker-based and markerless data were time-normalized and processed using Visual3D (v2021.03.2, C-Motion Inc., Germantown, MD). Marker-based data were filtered using a 4th-order dual-pass Butterworth filter with a lowpass cutoff of 6 Hz.(18) Segmental definitions used for marker-based anatomical model creation are described in Appendix B. For markerless data, Visual3D automatically creates all segments upon loading *Theia3D* data. *Theia3D* software uses a customized Visual3D anatomical model with pre-defined joint definitions that are not modified by an end-user, therefore, note that there are inherent differences in joint mobility constraints between anatomical models. The Visual3D marker-based and markerless anatomical models were used to calculate lower limb joint angles identical to prior comparisons of these approaches with healthy adults.(16) Joint angles were decomposed using a Cardan sequence X (lateral), Y (anterior), and Z (vertical). Joint angle time-series waveforms are reported between two consecutive heel strikes as % Stride determined by the algorithm described by Zeni et al. (2008).(19)

Following joint angle calculations, data were exported to Matlab (R2022a, The Mathworks, Inc., Natick, MA). Each joint angle time series was normalized by removing the best straight-line linear trend from the time series, using the ‘detrend’ function. The following discrete outcome variables were computed from joint angle data for both marker-based and markerless methods: root mean square (RMS), maximum peak (MAX), minimum peak (MIN), and range between the maximum and minimum peaks (RNG). The average of all trials was computed for each participant and used in statistical analysis.

### Statistical Analysis

All data were normally distributed according to Kolmogorov-Smirnov tests. Therefore mean, standard deviation (SD), 95% confidence interval (95% CI), and paired t-tests were used for comparisons between marker-based and markerless methods for noted discrete outcome variables. Cohen’s effect size and statistical power were calculated for each comparison using an *a priori α* = 0.05. Root mean square error (RMSE), Pearson’s coefficient of correlation (r), and coefficient of multiple correlation (CMC) were computed for time series waveform comparisons. All statistical analyses were performed in Matlab (R2022a, The Mathworks, Inc., Natick, MA).

## Results

The purpose of this study was to compare the kinematics of children with CP and individuals with chronic stroke during overground walking measured with marker-based and markerless motion capture systems using *Theia3D*. Few significant differences were observed between the two systems, suggesting this markerless technology could be a viable alternative for use in clinical settings. When possible, we discuss our comparisons in the context of Minimal Clinically Important Difference (MCID) and Minimal Detectable Change (MDC). Values at or below MCID or MDC suggest that any difference between the two systems is not great enough to affect clinical decision-making.

Root mean square values of joint angle variables had only two significantly different outcomes between marker and markerless systems (Table 2). Differences between the mean maximum joint angle measured by marker-based and markerless systems were minimal and below 4.5° (Appendix D). Differences between the mean minimum joint angle were also minimal and below 5° (Appendix E). Waveform comparisons from CMC and Pearson correlations had general trends of higher correlations in the sagittal plane joint angles than frontal and transverse (Appendix F).

At the trunk, mean differences of maximum and minimum joint angles between were less than 1° and not statistically significant amongst all planes. Discrete RMSE were between 2–4° for all planes, which resulted in large normalized RMSEs, particularly in the sagittal and frontal planes (Table 2). The total range of motion was also similar between the two systems with no statistical differences reported in our clinical populations.

At the hip, no significant differences were observed between systems in any anatomical plane for maximum joint angle of either limb or the minimum joint angle for the more affected limb. Mean differences in the sagittal plane (flexion/extension) were below an MDC of 4.69° and 4.01° shown for hipflexion and extension in stroke during stance and swing, respectively.(20) Transverse plane (rotation) of the more affected limb had a significant difference of 3.5° (Cohen’s *d* = 1.26, p = 0.017) for the minimum joint angle. The RMS for frontal plane motion of the hip was different between the two systems − 1.2° (Cohen’s *d* = 1.42, p = 0.026). However, RMSE for the more affected side was within 2.72°, and RMSE for the less affected side was smaller than that of 2.6° in healthy adults,(16) indicating that these results in neurological populations are near or within typical bounds of those in healthy adults.

There were no significant differences between the two systems at the knee, on either the more or less affected leg, in any of the three planes. The mean difference in minimum and maximum joint angles were less than the MCID for both knee sagittal plane range of motion of 8.48° for the affected side and 6.81° for the unaffected side(21) as well as the 6.43° and 5.25° MDC of kneeflexion and extension during stance and swing in chronic stroke gait, respectively.(20) The frontal plane knee angle showed insignificant difference, consistent with previous literature,(16) again demonstrating that the difference between the two systems in neurological populations is similar to that in healthy adults.

At the ankle, the mean difference in the sagittal plane for both the more affected and less affected limbs was well within MDC of 2.05° and 3.95° shown for ankle dorsi- and plantarflexion in stroke in stance and swing for chronic stroke gait, respectively.(20) The RMS in the frontal plane for the less affected limb was significant (−1.3° [Cohen’s d = 1.04] p = 0.043). However, the RMSEs for the sagittal, frontal, and transverse planes were all less than those of healthy adults found in previous work (6.7°, 8.0°, and 11.6°, respectively(16)). This indicates our results in neurological populations are within the bounds of those of healthy adults.

This subject was a Gross Motor Function Classification System (GMFCS) level 1, which implies minor issues with ambulation. Waveforms are normalized to 100% of a gait cycle defined as initial contact to initial contact. Shaded regions represent one standard deviation about the solid line which represent ensemble means.

This subject was 6.55 years post-stroke and used a right ankle foot orthosis and two-wheel walker. Waveforms are normalized to 100% of a gait cycle defined as initial contact to initial contact. Shaded regions represent one standard deviation about the solid line which represent ensemble means.

## Discussion

**Figure 1:**
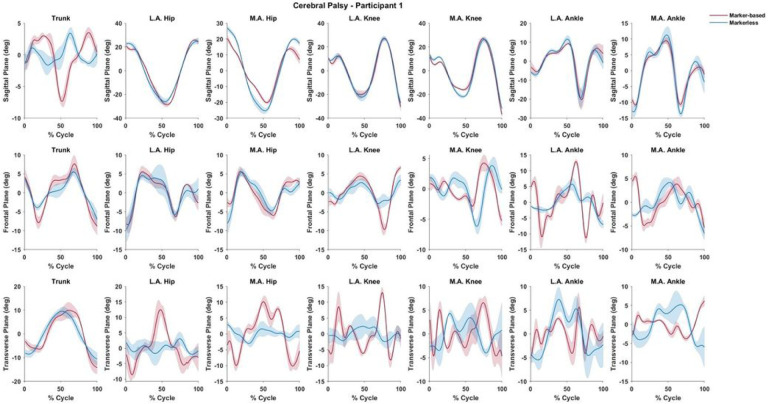
Time-series joint angle waveforms for a representative child subject with cerebral palsy.

**Figure 2:**
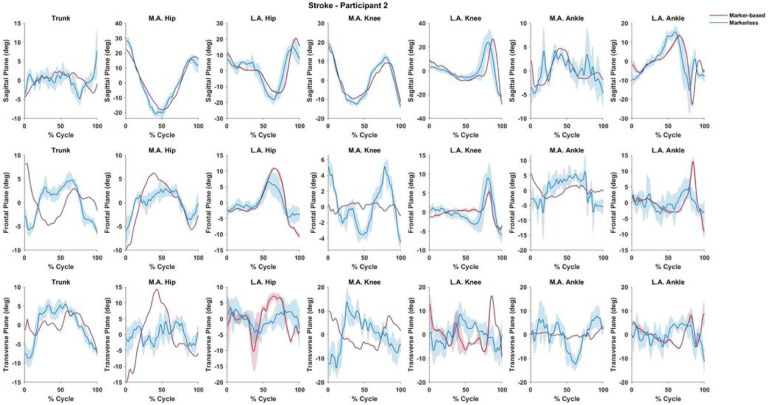
Time-series joint angle waveforms for a representative adult subject with chronic stroke.

The mean differences between systems may be challenging to interpret due to differences in approach between the two systems. This general variability in joint angle consistency between the two systems may be due to inconsistencies in the calculation of segment angle in the global coordinate system. Therefore, if there is error in the global segment pose, the error will be amplified in joint angle measurement. In addition, while few of our transverse plane (rotation) comparisons were significantly different, absolute and normalized RMSE values were consistently high in this plane. This is evident when reviewing comparative waveforms (Figs. 1 and 2) as the markerless waveforms for CP patients showed consistently poor agreement. Several studies have highlighted difficulties in accurately measuring tibial torsion and general internal/external rotation of the knee joint from marker-based approaches when compared to computed tomograph and goniometry.(22, 23) We speculate that this could be related to foot deformities and smaller anatomical features of the pediatric CP foot proving difficult for the machine learning algorithm to identify.

There are several potential sources of error that could influence joint angle measurement results in both systems. Marker-based systems are subject to operator error and inaccurate marker placement resulting in incorrect joint center estimation, as well as soft tissue or clothing artifact. These errors may lead to poor representation of anatomical landmarks and subsequent joint center locations, with amplified errors when calculating kinematics.(24) Marker placement error may contribute to up to 5° of error in lower limb joint angles.(25) Additionally, movement of soft tissue relative to bone during gait may translate up to 2.5 cm and rotate up to 8°, which introduces participant-specific errors in joint angle calculations.(26)

Markerless joint angle calculations may be affected by the training of the deep learning algorithm used to estimate pose, which is both a strength and weakness of the technology. This method also utilizes a frame-by-frame approach, which may introduce more noise than marker-based systems, but this noise may be overcome with the use of multiple measurements.(15) However, the sensitivity of the markerless motion capture system to participant-specific characteristics like age, sex, ethnicity, health condition, anatomical deformities, orthosis use, assistive device use, and clothing, as well as environmental factors, such as lighting, have yet to be fully tested.(16)

This study is not without limitations. This case study series included 6 total participants – 3 children with CP and 3 individuals with chronic stroke – both of which are largely heterogeneous populations. However, we attempted to address this limitation by including participants with varying levels of gait function. Two stroke participants utilized ankle foot orthoses and assistive devices, which may have influenced marker placement and marker visibility. In addition, the GMFCS III CP participant used a rear-walker

## Conclusions

This study demonstrates the accuracy and feasibility of the current *Theia3D* iteration for use in kinematic gait analyses of those with CP and chronic stroke. Our data show the differences between the two systems primarily fall within the relevant clinical measurement error for gait kinematics. These minor differences across a wide range of ages, heights, and adiposity suggests that *Theia3D* could be a suitable replacement for marker-based tracking of the trunk segment in clinical applications. This technology may prove a valuable tool in clinical settings where the practicality of a markerless motion capture system is necessary. Further investigations are warranted to determine if markerless techniques are an acceptably accurate method of capturing kinematic data with a variety of clinical populations in cases where the practical benefits of markerless data correction are warranted.

## Figures and Tables

**Table 1:** Participant demographics and clinical presentations. Gross Motor Function Classification Scale (GMFCS) is a clinical measurement of overall motor function in individuals with Cerebral Palsy. Assistive device use is also reported, including the use of Ankle Foot Orthoses (AFOs).

Participant	Pathology	Age (Yrs)	Height (m)	Weight (kg)	Sex	GMFCS	Time since stroke (Yrs)	Assistive Devices
CP1	Left hemiplegic	15.66	1.69	47.70	F	1		Left AFO
CP2	Left hemiplegic	6.99	1.22	31.28	M	3		Bilateral AFOs, Reverse Walker, Wheelchair at school
CP3	Left hemiplegic	8.08	1.36	38.40	M	2		Bilateral AFOs, Pushchair for primary mobility
Stroke1	Right hemiplegic	48.78	1.54	97.52	F		0.52	N/A
Stroke2	Right hemiplegic	68.84	1.60	67.59	F		6.55	Right AFO, 2-wheel walker
Stroke3	Left hemiplegic	66.44	1.74	83.46	M		7.57	Left AFO, single-point cane on right side

**Table 2:** Respective joint angle values for root mean square comparisons of the trunk and lower limbs. Bolded and * entries in the *p*-value column indicate a significant difference at the *p* = 0.05 level.

Side	Joint	Plane	Marker Mean (SD)	Markerless Mean (SD)	Diff. Marker – Markerless	Marker 95% CI	Markerless 95% CI	Cohen’s *d*	Power	*p*-value
-	Trunk	Sagittal	2.2 (1.2)	2.1 (0.5)	−0.1 (−4.6%)	−0.2–4.6	1.2–3.0	0.08	0.04	0.909
		Frontal	3.2 (1.9)	3.1 (0.9)	−0.1 (−3.1%)	−0.5–6.9	1.4–4.8	0.05	0.03	0.930
		Transverse	4.1 (3.2)	5.3 (1.5)	1.2 (29.3%)	−2.2–10.0	2.4–8.2	0.51	0.33	0.322
More affected	Hip	Sagittal	12.0 (4.7)	14.0 (4.2)	2.0 (16.7%)	2.8–21.0	5.8–22	0.43	0.25	0.291
		Frontal	3.2 (1.1)	2.9 (1.1)	−0.3 (−9.4%)	1.0–5.4	0.7–5.1	0.26	0.11	0.568
		Transverse	4.4 (2.3)	2.5 (1.1)	−1.9 (−43.2%)	−0.1–8.9	0.3–4.7	1.01	0.89	0.110
More affected	Knee	Sagittal	12.0 (5.2)	12.0 (5.0)	0.0 (0.0%)	1.8–22.0	2.2–22.0	0.00	0.03	0.999
		Frontal	2.5 (1.8)	3.4 (1.6)	0.9 (36.0%)	−1.0–6.0	0.3–6.5	0.53	0.36	0.183
		Transverse	3.7 (1.4)	5.4 (1.6)	1.7 (46.0%)	1.0–6.4	2.3–8.5	1.09	0.93	0.085
More affected	Ankle	Sagittal	5.8 (4.0)	5.3 (2.5)	−0.5 (−8.6%)	−2.0–14	0.4–10.0	0.13	0.05	0.788
		Frontal	2.9 (1.3)	3.0 (1.0)	0.1 (3.5%)	0.4–5.4	1.0–5.0	0.14	0.06	0.818
		Transverse	3.9 (3.4)	4.5 (1.3)	0.6 (15.4%)	−2.8–11.0	2.0–7.0	0.20	0.08	0.752
Less affected	Hip	Sagittal	13.0 (4.0)	13.0 (3.6)	0.0 (0.0%)	5.2–21.0	5.9–20.0	0.25	0.10	0.649
		Frontal	4.6 (0.9)	3.4 (0.8)	−1.2 (−26.1%)	2.8–6.4	1.9–4.9	1.42	0.99	0.026*
		Transverse	4.1 (1.4)	3.2 (0.6)	−0.9 (−22.0%)	1.4–6.8	2.0–4.4	0.83	0.74	0.220
Less affected	Knee	Sagittal	13.0 (3.2)	12.0 (3.3)	−1.0 (−7.7%)	6.7–19.0	5.5–18.0	0.11	0.05	0.581
		Frontal	2.8 (1.2)	2.6 (0.8)	−0.2 (−7.1%)	0.5–5.2	1.0–4.2	0.17	0.07	0.833
		Transverse	4.3 (1.9)	5.6 (2.6)	1.3 (30.2%)	0.6–8.0	0.5–11.0	0.56	0.40	0.431
Less affected	Ankle	Sagittal	7.9 (1.7)	7.4 (2.1)	−0.5 (−6.3%)	4.6–11.0	3.3–12.0	0.27	0.11	0.693
		Frontal	4.4 (1.7)	3.1 (0.7)	−1.3 (−29.6%)	1.1–7.7	1.8–4.4	1.04	0.91	0.043*
		Transverse	4.1 (2.1)	4.6 (1.3)	0.5 (12.2%)	−0.1–8.2	2.1–7.1	0.27	0.12	0.708

## Data Availability

The datasets used and/or analyzed during the current study are available from the corresponding author on reasonable request.

## Abbreviations

3D: Three-dimensional
CP: Cerebral Palsy
GMFCS: Gross Motor Function Classification Scale
AFO: Ankle-foot Orthosis
RMS: Root Mean Square
MIN: Minimum peak
MAX: Maximum peak
RNG: range between maximum and minimum peaks
CMC: coefficient of multiple correlation
